# Blickdiagnose für Fortgeschrittene

**DOI:** 10.1007/s00063-024-01166-4

**Published:** 2024-08-22

**Authors:** Laura Arheilger, Nicolas Müller, Mattia M. Müller, Christoph Camille Ganter, Rene Hage, Mace Schuurmans, Sascha David

**Affiliations:** 1 Institut für Intensivmedizin, Unispital Zürich, Rämistrasse 100, 8091 Zürich, Schweiz; 2 Klinik für Infektionskrankheiten und Spitalhygiene, Unispital Zürich, Zürich, Schweiz; 3 Klinik für Pneumologie, Unispital Zürich, Zürich, Schweiz

**Keywords:** *Scedosporium*, Transplantation, Immunsuppression, Mykose, Dermatose

## Falldarstellung

Wir berichten von einer 67-jährigen Patientin, die aufgrund einer COPD vor 4½ Jahren eine Lungentransplantation erhalten hat. Trotz adäquater Triple-Immunsuppression (Tacrolimus, Mycophenolat und Steroid) kam es zu einer humoralen Abstoßungsreaktion im Rahmen einer Rhinovirusinfektion mit CMV-Reaktivierung. Im Kontext dessen imponierte ein respiratorisches Versagen Typ I, welches letztlich auf die Intensivstation und zur invasiven Beatmung geführt hat. Bei nachgewiesenem donorspezifischem Antikörper (DQ2; MFI-7213) erfolgte eine Intensivierung der Immunsuppression inklusive Plasmapherese, Steroidstoß (3 × 1 g Methylprednisolon), IVIG und einer Plasmazelldepletion (Rituximab). Hierunter erreichten wir einen deutlichen Rückgang der Inflammation und im Verlauf eine Besserung der respiratorischen Funktion. Besonders kompromittiert war die Patientin zusätzlich durch eine schwere „ICU-acquired weakness“, die das Weaning komplizierte, sodass eine Tracheotomie erfolgte.

**Abb. 1:**
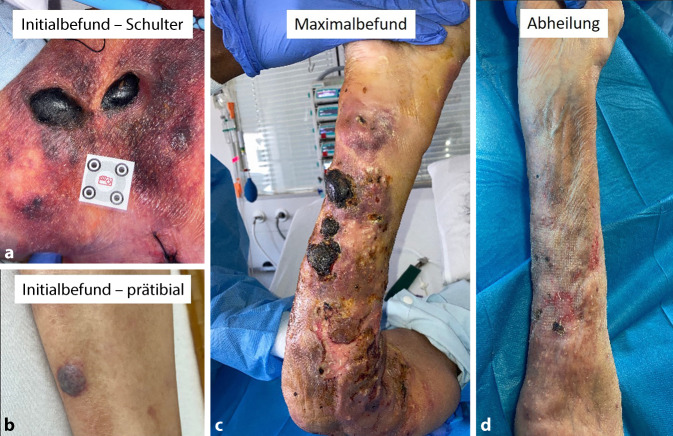
Klinischer Aspekt im zeitlichen Verlauf. **a** Initialbefund an Schulter und **b** Schienbein. **c** Maximalausprägung Arm (47 Tage) **d** Abgeheilter Befund (65 Tage)

In der 5. Woche bei uns fällt zunächst eine unspektakuläre eingeblutete Blase an der Schulter sowie am Schienbein auf (Abb. 1a,b). Innerhalb weniger Tage entwickeln sich multiple Läsionen in teilweise groteskem Ausmaß über den gesamten Arm mit einzelnen Ulzerationen bis auf die Muskulatur (Abb. 1c). Die Kollegen der Dermatologie gehen zu diesem Zeitpunkt von mechanisch bedingten eingebluteten Bullae aus und führten wegen des ausgeprägten Befunds doch eine Biopsie durch. Parallel kam es zu einer fulminanten Verschlechterung im Sinne eines septischen Schocks.

## Wie lautet Ihre Diagnose?

Überraschenderweise fand man bioptisch in der Tiefe der Dermis bereits in der PAS-Übersichtsfärbung massenhaft Pilzhyphen, die sich als *Scedosporium (Pseudallescheria boydii)* kultivieren ließen. Die Blutkulturen blieben steril, im Trachealsekret fand sich lediglich ein kolonisierter sensibler *Acinetobacter baumannii*, der nicht behandelt wurde. Aufgrund der raschen Progredienz des Befunds eskalierten wir die antimykotische Prophylaxe von Itraconazol unmittelbar auf eine duale Strategie aus hoch dosiertem Voriconazol und Micafungin und lokal Terbinafin. Um eine zielgerichtete Therapie der Antimykotika sicherzustellen, führten wir eine Empfindlichkeitstestung durch, in der sich Voriconazol und Anidulafungin mit niedrigen MHK (0,047 und 1,0 mg/l) als wahrscheinlich wirksam darstellten, wobei es für diesen Erreger keine etablierten Cut-offs gibt. Die alternative Kombination mit systemischem Terbinafin [1] war durch Interaktionen und Dialyse nicht durchführbar, weshalb wir uns auf eine topische Applikation beschränkten. Zudem wurde die Immunsuppression unter Inkaufnahme des erneuten Rejektionsrisikos reduziert (Tacrolimustalspiegel von 6 bis 8 auf 4 bis 6 μg/l, Halbierung Mycophenolatmofetil). Da Voriconazol ein starker Inhibitor von CYP3A4 und Tacrolimus dessen Substrat ist, wurden regelmäßige Spiegelmessungen durchgeführt, bei Tacrolimus täglich.

Zunächst wurde auch eine Amputation des Arms zur Reduktion der Pilzlast diskutiert (*„life before limb“*), was laut den Angehörigen jedoch strikt von der Patientin abgelehnt würde, sodass wir hiervon Abstand nahmen.

Bereits nach ca. 8 Tagen kam es zu einer unerwarteten Stagnation der Befunde mit Verbesserung über Wochen (Abb. 1d). Eine CT von Thorax/Abdomen zeigte keine Dissemination und im Tracheobronchialsekret wie auch in der Bronchiallavage ließ sich kein Pilzwachstum nachweisen. Bei verzögertem Aufwachen wurde im cCT jedoch ein mykotisches Aneurysma vermutet, dem wir letztendlich dem Patientenwunsch, keine Neurointervention durchzuführen, folgend nicht weiter nachgingen.

Leider entwickelte die Patientin zu einem späten Zeitpunkt (Tag 90 der Intensivtherapie) bei fast ausgeheiltem Lokalbefund einen erneuten refraktären septischen Schock im Rahmen einer *Klebsiella-pneumoniae*-Bakteriämie, sodass wir uns im Konsens mit der Familie für eine Therapiezieländerung entschieden und die Patientin verstarb.

**Differenzialdiagnostisch **kamen vor allem andere Schimmelpilze, deren Hyphen histologisch nicht von *Scedosporium* spp. unterscheidbar sind, infrage. Auch bakterielle Infekte, insbesondere durch Pseudomonaden, die durch Immunsuppression ebenso begünstigt werden, wurden gesucht. Auch wurden aufgrund der bullösen, z.T. eingebluteten Läsionen metabolische Erkrankungen, hereditäre Epidermolysen, Arzneimittelreaktionen, physikalisch-chemische Noxen, granulomatöse Erkrankungen, ein bullöses Pemphigoid und Neoplasien in Betracht gezogen.

Seit dem letzten Jahrhundert entwickelt sich eine starke Zunahme an Pilzinfektionen, was – neben der Zunahme an Antibiotikaresistenzen – als großes Problem des 21. Jahrhunderts betrachtet werden kann [2–4]. Nebst den zwei typischen Gattungen *Candida* spp. und *Aspergillus* spp. sowie endemischen Mykosen wie *Cryptococcus* spp. existieren auch bedeutsame weniger bekannte humanpathogene Gattungen. Eine Rolle spielt hierbei die wachsende Anzahl an stark immunsupprimierten Patienten im Kontext von Transplantationen und Autoimmunerkrankungen.

*Scedosporium* ist die asexuelle Form von *Pseudallescheria boydii* und ist weltweit in Böden und Gewässern anzutreffen. Der Erreger gelangt vor allem via Inhalation von Sporen, Aspiration von kontaminiertem Wasser oder durch Hautverletzungen in den Körper [5–7]. Dort kann er als lokalisierter Tumor wachsen, sich subkutan ausbreiten oder über die Blutbahn disseminieren [6].

Eine Manifestation tritt besonders häufig bei Neutropenie als invasiver Infekt und unter langjähriger Steroidtherapie auf, wenn eine gestörte zellvermittelte Abwehr oberflächliche Infekte begünstigt [6, 8]. Bei zystischer Fibrose ist *Scedosporium *spp. im Sinne einer Kolonisation das zweithäufigste Isolat der Atemwege [6]. Lokalisierte Infekte sind bei immunkompetenten Patienten seltener als bei *Aspergillus*, gewinnen aber an Bedeutung [5, 6].

**Diagnose:** *Scedosporium* Infektion unter intensivierter Immunsuppression

### Klinik.

Möglich sind viele unspezifische Symptome. Eine Infektion der Haut manifestiert sich oft als flächiger erythematöser Befund mit Papeln und Bullae mit nekrotischem Zentrum. In unserem Fall waren hier hämorrhagische Bullae zu sehen, die Beteiligung der Extremitäten ist typisch.

Pulmonale Symptome können einer Pneumonie mit Husten, Fieber, Sputum sowie pleuritischen Schmerzen entsprechen. Es kann eine Keratitis bzw. Endophthalmitis auftreten. In schweren Fällen kann es zur Dissemination mit septischem Schock und Multiorganversagen kommen [6, 7].

### Diagnostik.

Möglich ist der direkte kulturelle Nachweis auf Basis einer Biopsie, eines Punktats oder eines Abstrichs [6, 7]. Als molekulare Methode steht auch eine PCR mit Sequenzierung zur Verfügung. (FFPE; [6]). Mikroskopisch zeigen sich *Aspergillus*-ähnliche Hyphen mit nachweisbarem Zellwandpolysaccharid 1,3-beta-D-Glucan, die makroskopisch grau-braun watteartig anmuten [5, 6]. Im Falle einer Dissemination sind mehrheitlich positive BK zu erwarten [6]. Ein serologischer Nachweis ist bisher nicht verfügbar. Eine Dissemination sollte via CT gesucht werden und besteht, sobald ≥ 2 Körperregionen ohne Konfluenz betroffen sind. Besonders Hirnabszesse sollten bei immunsupprimierten Patienten ausgeschlossen werden ([6]; Infobox 1).

### Infobox 1 Diagnosesicherung

Gesicherte Diagnose: Hyphen histopathologisch aus Gewebe + positive Kultur

- Hyphen Histopathologisch + PCR/Sequenzierung (statt Kultur)
- Radiologie + Kultur
- Keine Klinik = nur Kolonisation
- [6]

### Therapie.

Es besteht eine geringe Empfindlichkeit gegenüber vielen antimykotischen Substanzen; keine Wirksamkeit hat das häufig prophylaktisch eingesetzte Fluconazol. Eine Empfindlichkeitsprüfung mit der Bestimmung der spezifischen MHK ist daher zur erfolgreichen Therapie essenziell. Falls eine chirurgische Sanierung des betroffenen Gewebes möglich ist (in unserem Fall Amputation des Arms), zeigt sich ein günstiger Einfluss ([9]; Tab. 1).

**Tab. 1:** Therapieoptionen

Gut wirksam	Intermediär	Unwirksam	Perspektivisch
Voriconazol Itra/Ampho [9]	Echinocandine [6, 9]	Fluconazol	Olorofim/Fosmanogepix Phase 2 abgeschlossen [9]
Posaconazol	Terbinafin („high MIC“; [6, 9])	–	–
Synergismus: Vori/Terbi [9] Vori/Mica [9]	–	–	–

Die Therapiedauer ist ungewiss; mindestens bis zur Regredienz der Symptome, im Falle einer persistierenden Immunsuppression kann eine jahrelange Therapie notwendig sein [9].

### Prognose.

Grundsätzlich besteht bei einer *Scedosporium*-Infektion eine sehr hohe Mortalität von 40 bis 100 % [9]. Dies erklärt sich auch durch die typischen Komorbiditäten der Patienten und deren Grad der Immunsuppression. Für die Prognose ist zudem die Möglichkeit einer Reduktion der Erhaltungsimmunsuppression bedeutsam – was zwangsläufig ein erhöhtes Rejektions- bzw. Rezidivrisiko von Autoimmunerkrankungen zur Folge hat. Außerdem spielt es eine Rolle, ob eine chirurgische Sanierung möglich ist und ob wirksame Antimykotika verfügbar sind [9].

## Fazit für die Praxis

- Bei immunsupprimierten Patienten müssen auch seltene, für ungeübtes Personal schwer zu erkennende Infektionen in Betracht gezogen werden.
- Neue Pilzinfektionen scheinen im Kontext altbewährter Immunsuppression und neuer Biologika immer häufiger aufzutreten.
- Fluconazol als häufige Prophylaxe ist gegen *Scedosporium *nicht wirksam.
- An die Austestung der Wirksamkeit/MHK muss bei seltenen Pilzinfektionen gedacht werden.
